# Emerging High-Frequency Ultrasound Imaging in Medical Cosmetology

**DOI:** 10.3389/fphys.2022.885922

**Published:** 2022-07-04

**Authors:** YaPing Tao, Cong Wei, YiMin Su, Bing Hu, Di Sun

**Affiliations:** ^1^ Department of Ultrasound in Medicine, Shanghai Institute of Ultrasound in Medicine, Shanghai Jiao Tong University Affiliated Sixth People’s Hospital, Shanghai, China; ^2^ Department of Ultrasound in Medicine, Kunming Fourth People’s Hospital, Kunming, China; ^3^ Department of Dermatology, Shanghai Jiao Tong University Affiliated Sixth People’s Hospital, Shanghai, China

**Keywords:** high-frequency ultrasound, medical cosmetology, dermatology, pathological scar, port wine stain

## Abstract

Cosmetic skin diseases are a part of many dermatological concerns brought up by patients, which negatively affect mental health and quality of life. Imaging technology has an established role in the diagnosis of cosmetic skin diseases by recognizing information on deep skin lesions. Due to the complex physiological and pathological nature of cosmetic skin diseases, the diagnostic imaging performance varies greatly. Developing noninvasive technology models with wide applicability, particularly high-frequency ultrasound (HFUS), which is able to achieve high-resolution imaging of the skin from the stratum corneum down to the deep fascia, is of great significance to medical cosmetology. To explore the great potential of HFUS in cosmetic skin diseases, a narrative review of literature from PubMed and Web of Science published between 1985 and 2022 was conducted. This narrative review focuses on the progression of HFUS imaging in medical cosmetology, especially on its promising application in the quantitative evaluation and differential diagnosis of cutaneous pathological scar, port wine stain (PWS), acne, skin aging, and other cosmetic applications.

## Introduction

With the improvement of technology and living standards, people pay more attention to cosmetic skin diseases, which deeply impact the patients’ quality of life from a functional, cosmetic, or psychological point of view (Han, 2019; Pirri et al., 2020). The deep understanding and clinical practice of skin repair and medical cosmetology derived from dermatology has developed into an independent medical field. An accurate assessment is fundamental for diagnosis and selection of therapeutic options, which needs support from convenient noninvasive imaging methods (Dill-Müller and Maschke, 2007; Heibel et al., 2020; Parashar et al., 2021). At present, the commonly used skin imaging technologies include skin photography, dermoscope, reflectance confocal microscopy, multispectral optoacoustic tomography, optical coherence tomography, and HFUS. Among them, skin high-frequency ultrasound (HFUS) can display and accurately measure not only the thickness of each skin layer but also the thickness and depth of deep lesions (Bakos et al., 2018; Lester et al., 2019; Schneider et al., 2019; Wan et al., 2021). Although HFUS application in medical cosmetology is increasingly valued, the wide and reliable application of HFUS imaging in medical cosmetology is still not yet recognized by dermatologists and radiologists. Moreover, the practical progress and systematic understanding of emerging ultrasound imaging in medical cosmetology are rarely reported. For these reasons, we review the progress of HFUS imaging in medical cosmetology, focusing on its promising potential in the quantitative evaluation and differential diagnosis of cutaneous pathological scar, PWS, acne, skin photoaging, and other cosmetic applications.

The major pathological changes involved in medical cosmetology include the morphology and distribution of blood vessels, changes in skin thickness, and specific changes in pigments, skin appendages, and epidermal structures. Conventionally, clinicians rely mainly on dermoscopy for a clear observation of the epidermal structure and dermatosis, confocal microscopy is frequently used in the diagnosis and evaluation of the efficacy of common cosmetic skin diseases, and multispectral optoacoustic tomography and optical coherence tomography can evaluate skin photoaging. The characteristics and indications of these technologies are listed in Table 1. (Kunzi-Rapp et al., 2006; Lee J.-N. et al., 2008; Koehler et al., 2008; Kuhn and Angehrn, 2009; Ardigo et al., 2010; Zhao et al., 2010; Lai and Xu, 2011; Lee et al., 2011; Kleinerman et al., 2012; De Pasquale et al., 2013; Chiang et al., 2014; Lallas et al., 2014; Lin et al., 2015; Sugata et al., 2015; Trojahn et al., 2015; Kim et al., 2016; Porto and Ozog, 2016; Scotto di Santolo et al., 2016; Heng et al., 2017; Manfredini et al., 2017; Su and Zhou, 2017; Sylwia and Krzysztof, 2017; Mustak et al., 2018; Ho et al., 2021).

**TABLE 1:** Comparison of the advantages and disadvantages among the conventional skin imaging technologies.

Technology	Advantage	Disadvantage	Applicable disease/purpose (in medical cosmetology)	References
Skin photography	Accessible	Unable to display the deep structures	To evaluate the severity and efficacy of volume, damaging skin diseases	(Dill-Müller and Maschke, 2007; Lee et al., 2011; Chiang et al., 2014; Fang et al., 2017; Heng et al., 2017; Mustak et al., 2018)
Dermoscope	To observe the fine outline and color of the lesion surface that is invisible to the naked eyes	Zero imaging depth; unable to display the deep structures	To diagnose and differentiate common skin diseases, and the changes in pigment and blood vessels after cosmetic treatment	(Lallas et al., 2014; Kim et al., 2016; Porto and Ozog, 2016; Su and Zhou, 2017)
Reflectance confocal microscopy	To obtain cell level imaging	Only detecting the superficial part of the dermis	To diagnose and differentiate vascular skin diseases, pigmented skin diseases, and acne, and evaluate the curative effect	(Ardigo et al., 2010; Lai and Xu, 2011; Lin et al., 2015; Manfredini et al., 2017)
Multispectral optoacoustic tomography	To study the metabolic process at the single cell level	Scanning depth of 200–300 um; unable to show deep lesions	To measure the morphology of normal human skin, characterize skin aging, study skin pharmacokinetics, evaluate the efficacy of medical skin care products	(Lee J.-N. et al., 2008; Koehler et al., 2008; Sugata et al., 2015)
Optical coherence tomography	Axial resolution up to 1.0–15.0 μm, can clearly show the morphology of the skin epidermis and dermis	Unable to achieve cell level imaging, or display the variability of deep disease over 2 mm	To monitor the microscopic changes such as photoaging and scar treatment and evaluate the effect of laser treatment	(Kunzi-Rapp et al., 2006; Zhao et al., 2010; Trojahn et al., 2015)
High-frequency ultrasound	To display full-thickness skin tissue and deep lesions	Influenced by age, microcirculation changes, edema	To measure the thickness of the normal skin and skin lesions, and monitor the efficacy of laser treatment, minimally invasive treatment and injection cosmetology	(Kuhn and Angehrn, 2009; De Pasquale et al., 2013; Scotto di Santolo et al., 2016; Sylwia and Krzysztof, 2017)

Given the complexity of the patient’s clinical needs, although various skin imaging technologies have specific advantages and uses, no single technology can cover all of the uses of medical cosmetology, especially regarding the meticulous functions such as the meticulous evaluation of the recovery state after cosmetic surgery. Since HFUS can achieve a balance between resolution and depth, it is particularly suitable for evaluating the extent and depth of deep skin lesions. In recent years, HFUS imaging has been widely used in the differential diagnosis and therapeutic evaluation of skin neoplasia, skin diseases, and inflammatory skin diseases, and thus can be used experimentally and clinically during medical cosmetology applications. In recent decades, based on the similar pathological characteristics and clinical needs of the above skin neoplasia, the application of HFUS in cosmetic medicine has also been proposed and has gradually received attention. Therefore, this review summarizes the following special application scenarios of HFUS images:

This is a narrative review. PubMed and Web of Science were searched for articles published between 1985 and 2022. The search terms were ultrasound, medical cosmetology, dermatology, cutaneous pathological scar, PWS, acne, and skin aging. Articles reporting HFUS imaging of medical cosmetology were selected. Articles focusing on other diagnostic methods or ultrasound therapy for skin diseases were excluded.

### The Basic Principles of High-Frequency Ultrasound Imaging

An ultrasound wave is sound with frequencies of over 20 kHz that the human ear cannot hear. Ultrasonic imaging technology is based on the properties of reflected sound waves through tissues. Ultrasound signals are emitted and received by the transducer after reflection in the tissues, the sonogram is depicted on the monitor in brightness mode. The echogenicity was evaluated by comparing it with the surrounding normal tissues and was described into three types: hyperechoic, isoechoic, and hypoechoic. HFUS is the basis for other imaging technology, including color and power doppler, ultrasound elastography, contrast enhanced ultrasound and so on. Doppler ultrasound is based on the principle that the transducer and the reflector of the sound wave are moving with respect to each other (Ho et al., 2021). Color and power doppler are used to evaluate the vascularity. Ultrasound elastography is a technique used to get an image of the strain on a tissue imposed by a force. It can be used to calculate the elasticity of the tissue (Kleinerman et al., 2012). Elastic imaging has some interesting applications in medical cosmetology (Lung et al., 2020; Patwari et al., 2022). Meanwhile, the application of artificial intelligence (AI) is accelerating in medical cosmetology and has the potential to transform dermatology workflows with its applications in image recognition through utilizing machine learning, convolutional neural networks, and so on. It has great potential for patient care in medical cosmetology, particularly in improving the sensitivity and accuracy of skin lesion screening (Hogarty et al., 2019; Daneshjou et al., 2022).

## Application of High-Frequency Ultrasound in Cutaneous Pathological Scars

### Showing the Histological Level of Cutaneous Pathological Scars

Cutaneous pathological scars are fibrotic lesions that grow continuously to invade the adjacent skin and are erythematous, itchy, and painful. Two of the advantages of HFUS over dermoscope, reflective confocal microscopy, and other skin imaging technologies to observe the skin surface features, are its larger tissue penetration depth and its ability to clearly display pathological scars at 14–50 MHz at the histological level. The epidermis, dermis, and subcutaneous tissue are shown as a hyperechoic line, a hyperechoic band, and a hypoechoic layer, respectively (Dill-Müller and Maschke, 2007). Ultrasound elastography is a technique used to create an image of the strain on a tissue imposed by a force and is a logical addition to HFUS. Aya R et al. (2014) reported their experience of keloids’ evaluation with ultrasound elastography. In their study, the immature elevated keloid was presented as a hypoechoic area in HFUS, while the mature flattened keloid had a similar echo signal as normal skin. Besides, the evaluation of keloids using ultrasound elastography allows for a noninvasive examination to objectively measure the stiffness of the lesions, and it seems to be a potential tool to assess skin scars. Agabalyan et al. (2017) used 20 MHz HFUS to study the relationship between the thicknesses of 10 burn scars and histological controls and found weak correlations among the epidermis, dermis, and total thickness. One of the reasons is the partiality of the results caused by insufficient ultrasound penetration. It is recommended that before its clinical use and research, we optimize two-dimensional ultrasound and select the right frequency probe to achieve good display results. Besides, Pirri et al. (2020) used HFUS imaging in the diagnosis of the cause of scar pain, indicating that the alteration of superficial fascia in thickness and echo characteristics is the pain generator.

### Assisting in Diagnosis and Identification of Different Types of Cutaneous Pathological Scars

**FIGURE 1:**
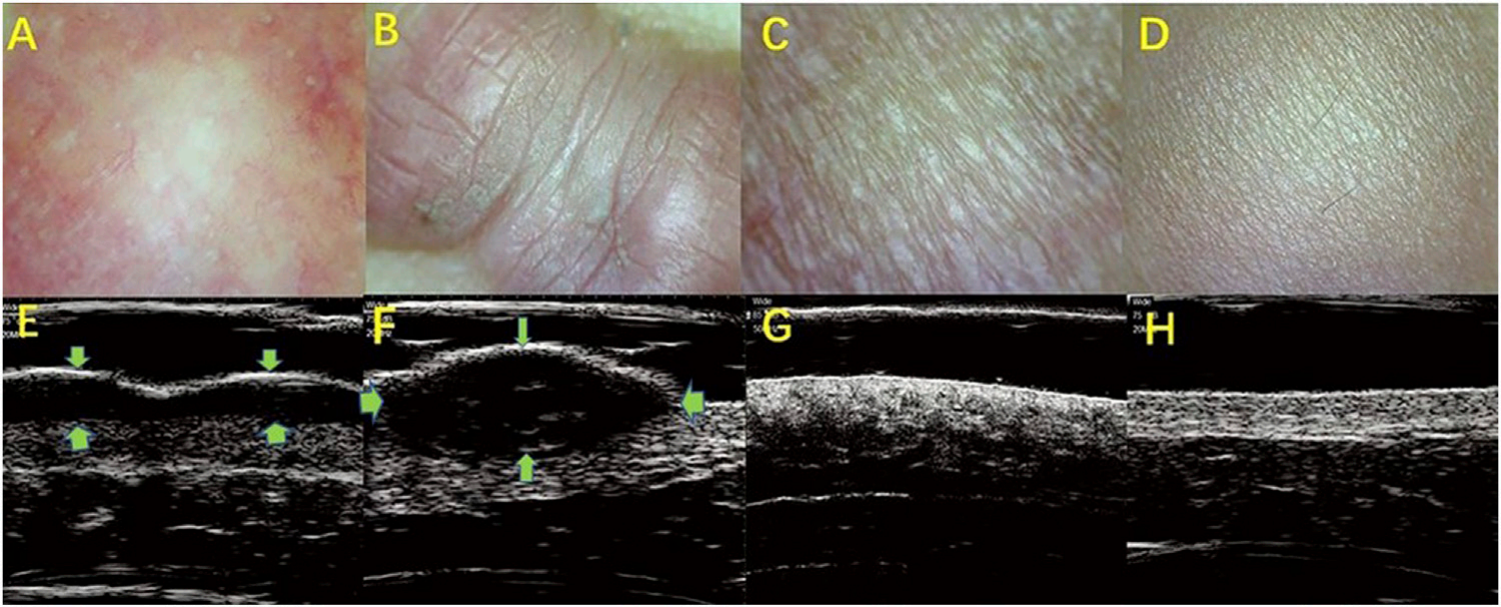
HFUS identifying the different types of skin scars. **(A,E)**: Hypertroplic scars; **(B,F)**: Keloids; **(C,G)**: Atrophic scars; **(D,H)**: Normal skin. **(A–D)**: The optical characteristics of various scars observed by skin microscopy; **(E–H)**: audio-visual characteristics of scar skin detected by 20 MHz HFUS.

Cutaneous pathological scars are common skin fibrotic lesions and mainly include hypertrophic scars and keloids. At present, the clinical scales used for scars and experience-based assessments are limited by their subjectivity, low repeatability, and other shortcomings. HFUS can objectively obtain the scar thickness, echo, morphology, blood flow, and other information and is a noninvasive method that provides more information. The combined clinical and radiological assessment is very helpful in assessing the scars and comparing the efficacy of different treatment modalities (Elrefaie et al., 2020). The researcher selected 48 scar patients and divided their scar categories into hypertrophic scar, keloid, and atrophic scar. With 20 people with normal skin as controls, 20 MHZ HFUS was applied to different types of scars. The audio-visual images of the normal skin found that the ultrasound images of different types of scar tissues had certain characteristics. A hypertrophic scar was characterized by a striped low-echo region in the upper dermis, and keloid was concentrated in the elliptical low-echo area under the epidermis. The skins of hypertrophic scars and keloids are significantly thicker compared with atrophic scars and normal skin, while the dermis and subcutaneous tissue boundary of atrophic scars is unclear (Figure 1). The results show that HFUS of skin can assist in the clinical diagnosis and identification of various scar tissues and can quickly, effectively, and objectively distinguish between scarring and hypertrophic scars (Wang et al., 2020).

**FIGURE 2:**
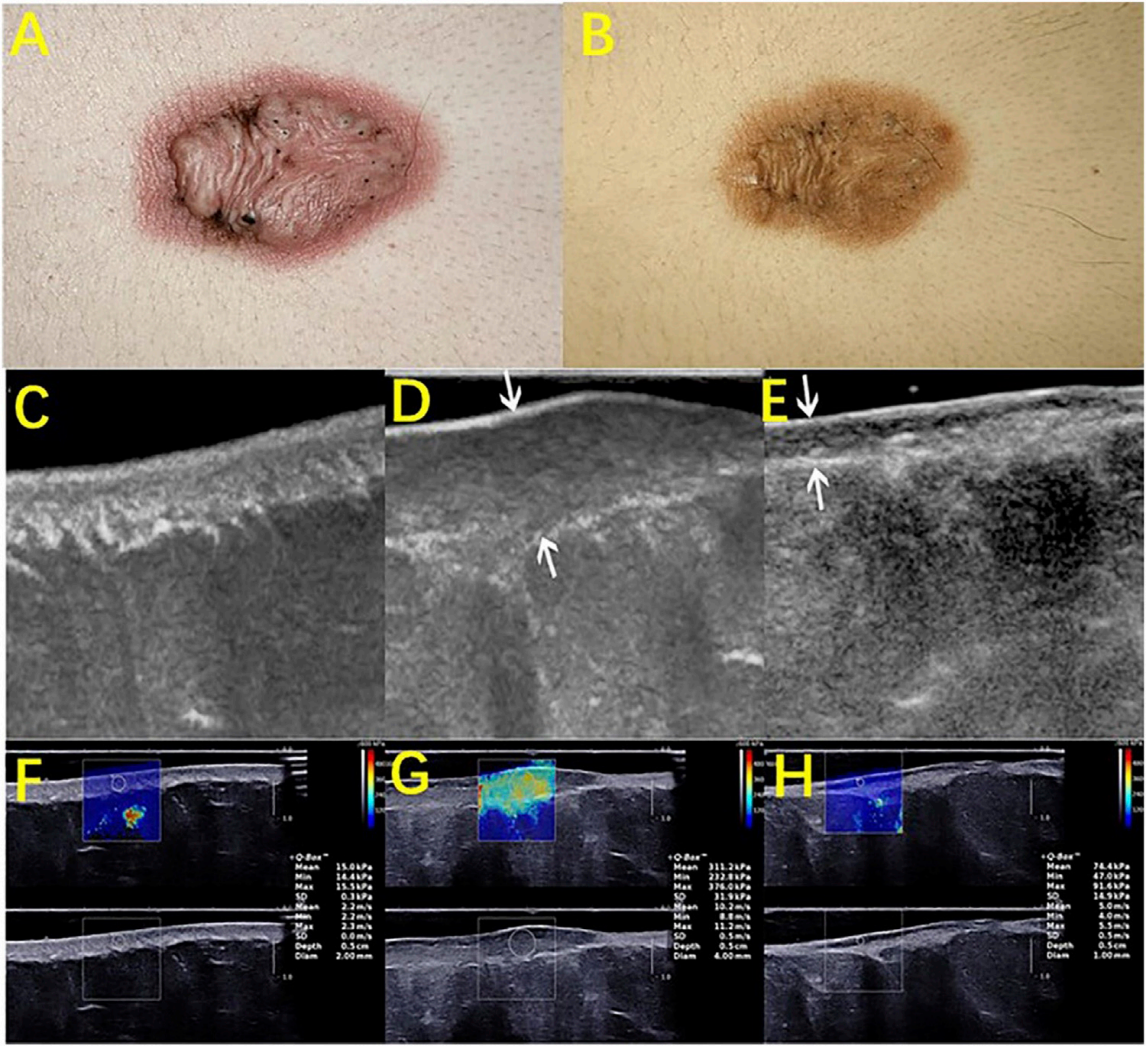
HFUS assessing the therapeutic efficacy of scars. A keloid before **(A)** and after **(B)** intralesional steroids injection. B-mode ultrasound images (longitudinal) of normal skin **(C)**, pretreated keloids **(D)**, and post treated keloids after an intralesional steroid injection **(E)**. **(F)**, **(G)**, and **(H)** are the corresponding elastography images of **(C)**, **(D)**, and **(E)**, respectively. Images **(A)** to **(H)** were captured from the same patient. Arrows in images **(D)** and **(E)** showed an obvious reduction in the scar thickness after treatment. The depth × width of the images **(C–E)** were 1.5 × 2.25, 1.6 × 2.4, and 1.5 × 2.25 (cm), respectively. As shown in the images, the quantitative elasticity values, including Young’s modulus and shear wave velocity, decreased after treatment.

**FIGURE 3:**
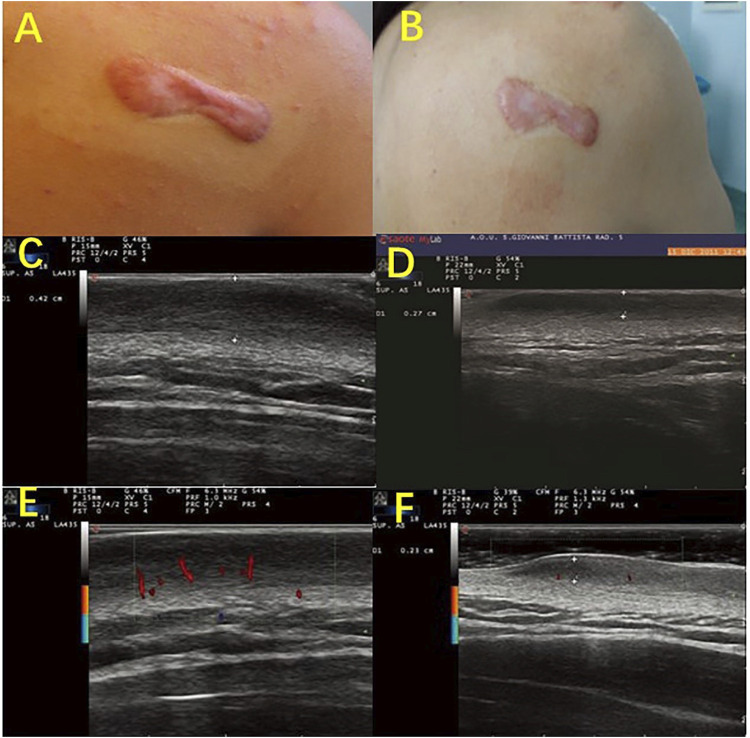
Color-Doppler ultrasonography evaluates the characteristic vasculature of scars. Photography of a scapular keloid before treatment **(A)** and after 1 month of treatment with a Pico device **(B)**. B-mode Ultrasound images of the keloid before treatment **(C)**, after 1 month of treatment **(D)**, and after 2 months of treatment with a Pico device **(F)**, show that the keloid thickness was reduced from 0.42 to 0.27 cm and then to 0.23 cm. Color-Doppler ultrasound images showed palisade vessels in the keloid before treatment €, and palisade vessels disappeared after 2 months of treatment with a Pico device **(F)**.

Cutaneous pathological scars are caused by an incorrect regulation of wound healing beyond the edge of the original wound, which rarely subsides over time and can lead to disfigurement and physical injury, resulting in psychological stress and a decline in quality of life. Among the various methods of scarring treatment, the evaluation of the scarring efficacy is of great concern. Studies that evaluated the use of HFUS to observe the improvement in scars found that the low echo disappeared in the center area of the scar, as the echo gradually increased to a level similar to the surrounding dermis, and the echo uniformity increased slowly (Reinholz et al., 2016; Schwaiger et al., 2018). Huang SY used HFUS combined with shear wave elastic imaging (SWE) to evaluate the efficacy of scar and found that the scar thickness and hardness were low after treatment and that the thickness was almost consistent with normal skin (Figure 2) (Huang et al., 2020). Moreover, the color doppler method that is used to observe the changes in the blood flow before and after scar pressure therapy found that the internal blood flow signal was significantly reduced or even disappeared after treatment (Figure 3). It has been suggested that HFUS, as a new noninvasive method, has great potential in evaluating the clinical efficacy of scars (Fraccalvieri et al., 2013; Gamil et al., 2020).

## Application of Skin High-Frequency Ultrasound in Port-Wine Stain

### Measuring the Thickness of Port-Wine Stain

Port-wine stain (PWS) is a benign capillary malformation that commonly occurs in the head and neck. From an imaging perspective, the lesions of the skin layer are mainly affected by PWS, and the CT and MRI manifestations are nonspecific. HFUS can clearly show the characteristics of the skin epidermis, dermis, and subcutaneous tissue anatomy after erythematous damage and can measure the thickness. Ni et al. (2016) detected the relationships of the skin thickness with age, location, sex, and other factors in children with PWS, and they measured and compared the thicknesses of normal skin and skin lesion using 20 MHz HFUS. The results showed significant differences in the skin thickness among the different parts of the skin, indicating that the skin lesion thickness in children with PWS could be accurately measured by HFUS. Gan et al. (2017) used 20 MHz HFUS to measure the thickness of 30 children with PWS before and after a course of pulsed dye laser treatment. An analysis of the measured results found that the thickness of PWS was thinner after the treatment. It is suggested that HFUS can accurately measure the skin thickness of normal skin and skin with PWS lesions (Gan et al., 2017).

### Distinguishing Between Normal Skin and Port-Wine Stain Skin After Skin Damage

**FIGURE 4:**
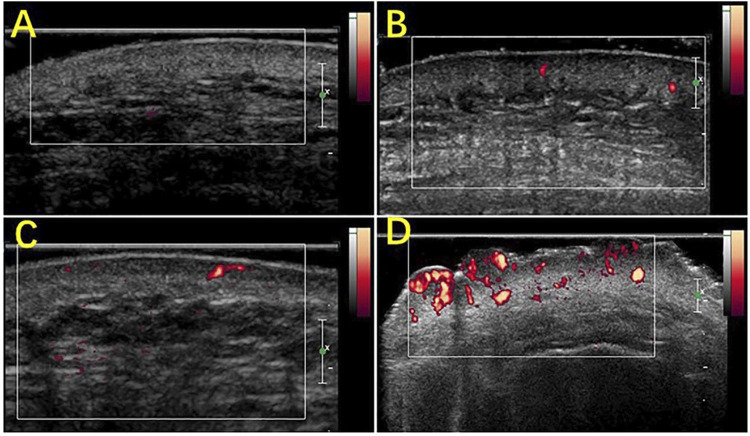
Scores of the blood flow signals in PWS lesions with Power Doppler ultrasound. **(A)** Grade 0, absence of color signals in a pink-type lesion; **(B)** grade 1, mild color signals in a thickened-type lesion; **(C)** grade 2, moderate color signals in a thickened-type lesion; **(D)** grade 3, marked color signals in a nodular-type lesion.

PWS patients have a light pink to dark purple skin tone, and the lesions can be divided by their clinical characteristics into four types: pink, purple, thickened, and nodular. HFUS was combined with (shear wave elastography) SWE to quantitatively evaluate 195 PWS patients (238 cases of skin loss) in comparison with normal skin groups. The dermis hypoechogenicity, thickness of thickened skin, and skin power doppler (PD) signal grades were all significantly higher in all PWS-type groups than in the normal-skin group. The thickened skin and skin PD signal grades in the nodular-type group and the thickened-type group were significantly thicker and higher, respectively, than those in the pink-type group and the purple-type group. The PD signal grades in the purple-type group were significantly higher than those in the pink-type group (Figure 4), suggesting that ultrasound can be used as a potential quantitative assessment tool to categorize erythematic skin loss (Tang et al., 2019).

### Quantitative Evaluations of the Efficacy of Photodynamic Therapy

PWS, as capillary malformations of the skin that are typically present at birth, can lead to a decrease in the quality of life and can increase the psychological burden and risk of depression. At present, the common treatment method for PWS is photodynamic therapy. Evaluation of the treatment effect of port wine stain used to mainly depend on standardized photos for the comparison of visual scores before and after treatment, but such evaluation lacks objective quantitative indicators. HFUS can observe the changes in audio-visual maps for the skin layer thickening at the skin loss area secondary to PWS, the increase in the blood flow signal and the reduction in the echo of the partial skin layer, which can quantitatively evaluate the efficacy of light power therapy for PWS. For instance, 72 PWS patients (90 cases of skin loss) who had undergone photodynamic treatment were assessed quantitatively by ultrasound imaging, including dermal low echo, skin thickness changes, and PD signal grading, which showed that skin thickness change was the best indicator to assess the efficacy (Tang et al., 2019). Tang et al. (2021) selected 60 PWS patients and used HFUS to compare the thickness and blood flow grading changes before and after treatment of skin loss thickening. It was found that for patients with a visual score of 0 points, the blood flow classification was not significantly different before and after the treatment of skin loss. Traditional visual scores may be associated with a poor response to photodynamic therapy and treatment termination, but HFUS identified a decrease in the skin damage thickness of such patients, indicating that HFUS can be more sensitive to the photodynamic therapy response than traditional visual scores. HFUS is expected to be a quantitative evaluation tool for the efficacy of photodynamic therapy for PWS.

## Application of High-Frequency Ultrasound in Acne

### Effective Diagnosis of Different Types of Acne

**FIGURE 5:**
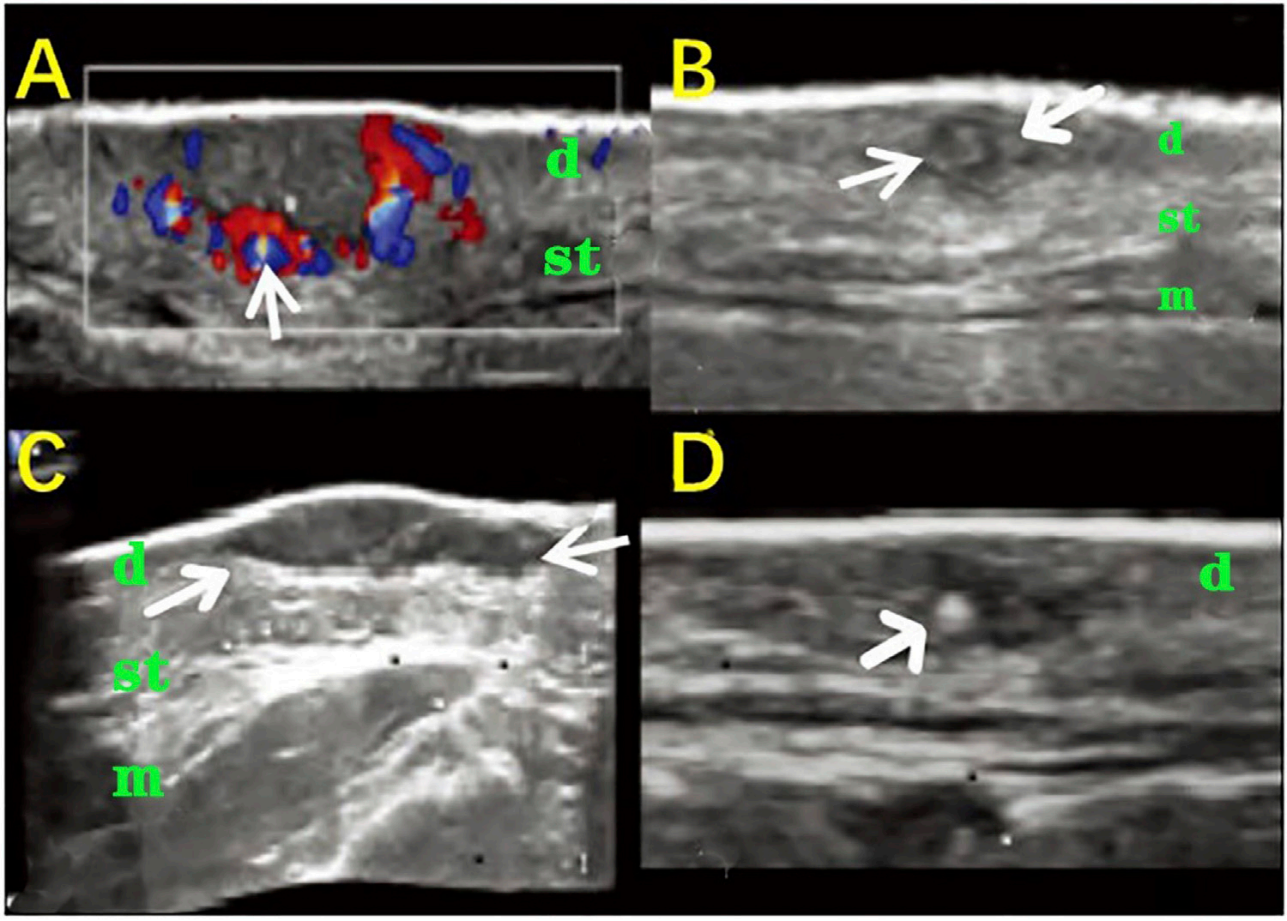
HFUS in identifying the different types of acne. **(A)** A false cyst-type dermis and subcutaneous tissue within a low-echo nodule; the arrow indicates the rich blood flow around the nodule; **(B)** The hair follicle type between the two arrows shows a slightly tilted low echo across the dermis; **(C)** A fistula type low-echo structure of the belt between the two arrows is located in the dermis and subcutaneous tissue layer; **(D)** The calcification arrow shows the dot calcification stove in the dermis. (d: dermis, st: subcutaneous tissue, m: face muscle).

Acne is an inflammatory skin disease common in young adults that mainly affects the skin hair follicle gland units. Acne can lead to post-inflammatory pigmentation and permanent scarring, which seriously affect the physical and psychological health of patients. An acne diagnosis mainly depends on the dermatologist’s physical examination. A touch diagnosis makes it difficult to obtain deep information on the lesions, especially the internal morphological structure. Acne can sometimes be found on visual examination and contact can be found, which can increase the difficulty of diagnosis and may even lead to treatment failure. Therefore, the precise location of acne, the type of judgment, and understanding the immersion depth are the keys to effective treatment and to obtaining a reduction in the recurrence, so there is an urgent need for a more accurate diagnostic tool. Wortsman et al. (2014) studied 245 acne lesions in 20 patients using HFUS and found that the acne ultrasound abnormalities included pseudocysts, folliculitis, fistulas, and calcinosis. Tan et al. (2018) conducted skin ultrasounds on 504 lesions in 40 acne patients, summarized their ultrasound characteristics, and evaluated the light, medium, and severe conditions in comparison with the judgment of dermatologists. The results demonstrated that the acne images were mainly shown in the form of fake cysts, hair follicles, fistulas, and calcifications (Figure 5), which is in line with some previous studies. In addition, ultrasound physicians and dermatologists have consistent judgments for mild patients, but their judgments for moderate and severe patients are significantly different. It is suggested that HFUS of the skin clearly shows the shape and type of acne and assists in determining the severity of the condition.

### Assessment and Prediction for the Therapeutic Effects in Acne

HFUS can also assess the efficacy of lasers in treating acne. For the first time, Naouri et al. (2011) used HFUS to monitor the efficacy of CO_2_ dot matrix laser subtreatment for skin acne and determined the dynamic change process in 104 cases of 24 skin acne patients after dermatological treatment. The dermis was significantly thickened after treatment. They believe that lasers promote collagen recombination, extracellular matrix reconstruction, and skin water cooperation.

Moreover, the impacts of different factors on the therapeutic effect were analyzed, which showed that the therapeutic effect was significantly affected by age and was more effective on young skin than on older skin. Fibroblasts in younger patients are more sensitive to heat and ablation stimuli, so early treatment can yield better results, indicating that older skin needs higher parameter settings. In addition, skin thickness also affects the therapeutic effect, and thin skin can be more effectively treated than thick skin. When the parameters and spore diameters were similar, thin skin was stimulated throughout the thickness range, while thick skin was only irritated on its surface, suggesting that additional settings were required for the optimal treatment of thicker skin. In summary, HFUS imaging can objectively assess the efficacy of CO_2_ subsurface laser therapy, but since the initial skin thickness measurement is needed to predict the efficacy, the skin thickness is an important influencing factor on the treatment response (Naouri et al., 2011; Malinowska et al., 2021).

## Application of High-Frequency Ultrasound in Skin Aging

### The Use of High-Frequency Ultrasound to Assess, Quantify, and Classify Skin Aging

**FIGURE 6:**
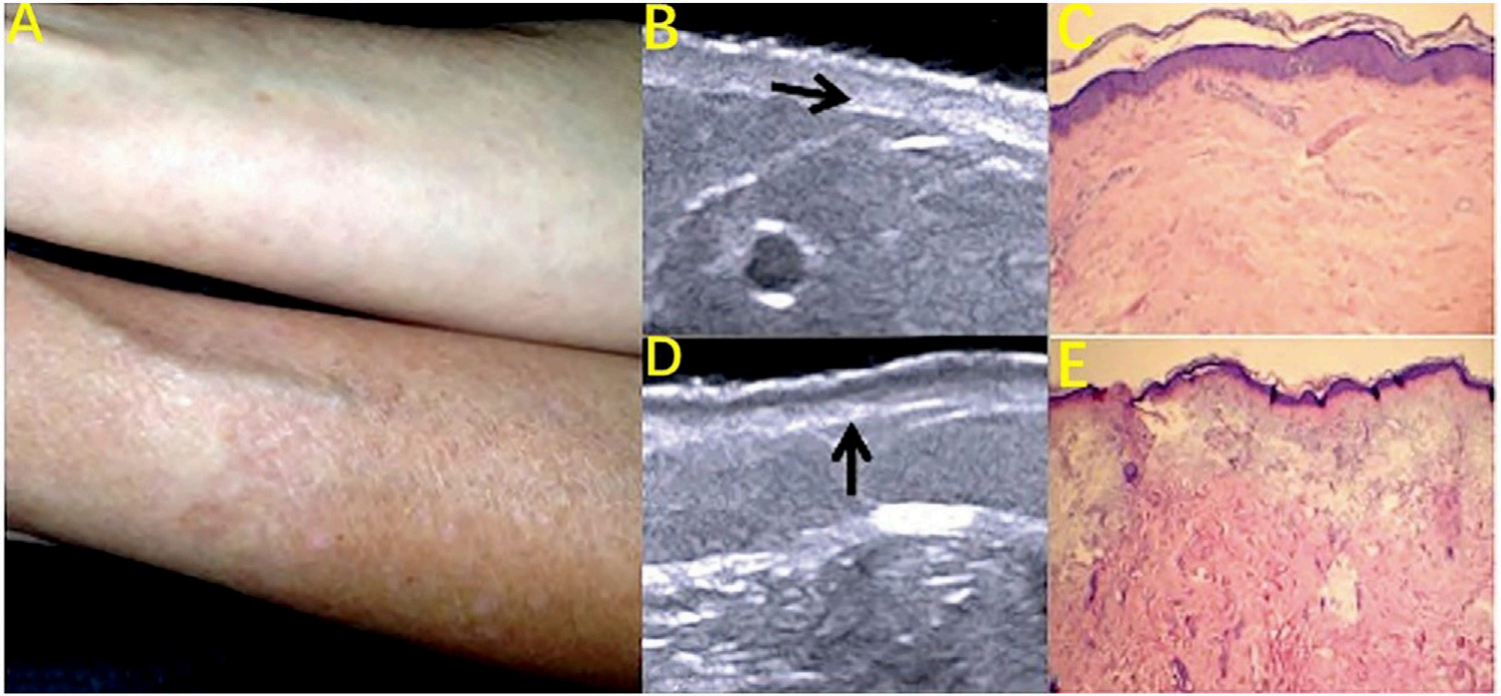
Skin photoaging: subcutaneous low-echo band (SLEB) on HFUS images. **(A)** Photography of the ventral and dorsal areas of the forearm; **(B)** Decreased dermis echogenicity (arrow) on HFUS image. **(C)** Discrete degeneration of collagen fibers. Hematoxylin and eosin (H and E) staining (10X). **(D)** SLEB (arrow) on the HFUS image. **(E)** Solar elastosis. H and E staining (10X).

Skin aging includes chronological aging and skin photoaging. The main histological features of chronological aging are dermal thinning, reduced collagen, elastic fibrosis, and less hydration. These histological changes are consistent with the decrease of echogenicity in the upper dermis on HFUS imaging, which is directly related to the density of the extracellular matrix. The tissue pathology changes associated with skin photoaging are solar elasticity, collagen structural changes, and glycoamine polysaccharide accumulation. These changes are reflected as a subcutaneous low-echo band (SLEB) in ultrasound images (Figure 6) (Sandby-moller and Wulf, 2004; Sandby-Møller et al., 2004; Barcaui et al., 2016). As a noninvasive method of evaluation, HFUS can clearly observe the structure of skin layers and can evaluate, quantify, and classify the aging of the skin (de Rigal et al., 1989; Barcaui et al., 2016). The main indicators are the skin thickness, density, echo, and the epidermis low-echo band (Wang et al., 2021). Lee HK et al. found a high correlation between the skin dermis density and the skin roughness as measured by Dermascan C HFUS diagnostic instruments, suggesting that the skin roughness can be more accurately assessed by measuring the dermis density with HFUS (Lee H. K. et al., 2008). de Rigal et al. (1989) found that the thickness of the SLEB identified by skin ultrasound was positively correlated with age and was thicker on the back side of the forearm than on the abdominal side, suggesting that SLEB can indicate and reflect skin photoaging. Gniadecka M also suggested that the appearance of low-echo bands under the epidermis was due to the epidermal nipple elasticity and relaxation and should be based on ultrasound imaging using the SLEB as an indicator of skin light aging noninvasive measurement. These studies indicate that the thickness of the SLEB can be measured by HFUS to assess the degree of skin photoaging (Gniadecka, 2001).

### Assessing the Effectiveness of Improved Skin Photoaging Treatment

Several studies have demonstrated the efficacy of local vitamin C therapy in increasing collagen production, reducing UV radiation-induced oxidative stress, and enhancing skin remodeling (Fanian et al., 2013; Addor et al., 2018). When HFUS was used to assess the skin of patients during 60 days of local vitamin C treatment, the echo of the epidermis and dermis increased after 40 days and was greater after 60 days (Diana et al., 2015). Ultrasounds using 15 and 20 MHz were also used to assess the effectiveness of oral micronutrient supplements in maintaining skin quality by measuring the skin thickness and density. The thickness was calculated directly from a region of interest determined in the HFUS images. The density was described as the mean luminance of the region of interest on the grayscale HFUS images (Fanian et al., 2013). The skin aging was improved after 3–4 months of oral micronutrient supplements, and HFUS showed a significant increase in the skin thickness (Diana et al., 2015). It is suggested that HFUS can be used to observe the efficacy of improving the skin aging treatment and is considered an effective tool for assessing skin photoaging. However, the reliability and correlation of relevant ultrasound parameters in determining the histological changes and dermis composition need further exploration (Wang et al., 2021).

## Application in Other Areas of Medical Cosmetology

### Detection of Common Cosmetic Fillers

Pure hyaluronic acid (HA) presents as small anechoic pseudocyst structures on HFUS images that ordinarily decrease in size in a short space of time, usually 3–6 months. HA mixed with lidocaine commonly appears as inner echoes within the pseudocyst structures and lasts 3–6 months. However, high-density HA is shown as small to medium-sized anechoic pseudocyst structures presenting with some echoes. High-density HA deposits decrease in size slowly, and the effects last more than 2 years. Oval hypoechoic solid nodules with clear or unclear boundaries may be detected in or around the high-density HA injection sites owing to the development of local inflammation and granulomas. Pure silicone presents as oval anechoic lacunar areas, which maintains its shape or size over time. In comparison, silicone oil is shown as hyperechoic deposits with a posterior acoustic reverberation artifact. This blurry white pattern of silicone oil is called “snowstorm.” HFUS can trace the mixed formulations of pure silicone and oily silicone, which can also result from the merging of pure silicone with hypodermal fatty tissues after a period of time. On HFUS images, polymethylmethacrylate appears as hyperechoic dots with a mini-comet tail posterior artifact. Calcium hydroxyapatite presents as hyperechoic deposits with a posterior acoustic shadowing artifact. Polyacrylamide gel is shown as anechoic oval pseudocyst structures, which commonly do not change their size or shape for at least 18 months. When patients do not know what substance was used for the previous filling, a skin HFUS can be used to detect the common cosmetic fillers and anatomical effects and monitor the longevity (Wortsman, 2015).

### Ultrasound-Guided Injection Therapy

Ultrasound is a real-time, noninvasive visualization tool that accurately guides the drug to the target area in real time and is more accurate than the traditional direct injection method, thus improving the success rate and reducing the development of complications caused by improper injection. Zhang JQ used ultrasound-guided drug injections to treat scarring and monitored the level of drug injections and the scope of the drug dispersion in real time (Zhang et al., 2017). Other studies have shown that ultrasound can prevent the formation of scar ulcers by injecting the guided drug into the scarred skin in real time (Dinh Huu et al., 2019). In addition, Thompson et al. (2015) reported that multiple injections of steroid treatment of distinguishing keloids can be ultrasound-guided to distinguish between injection keloids with mature areas and immature areas. In other words, the therapeutic dose to the immature area is higher than that to the mature area in order to achieve precision treatment. By using the HFUS color Doppler function to correctly identify the venous structure, researchers can visually guide the injection of foam sclerosis to treat venous ulcers in the lower extremities, which can achieve obtain good healing effects and low return rates (Lloret et al., 2015).

### Monitoring of Complications

**FIGURE 7:**
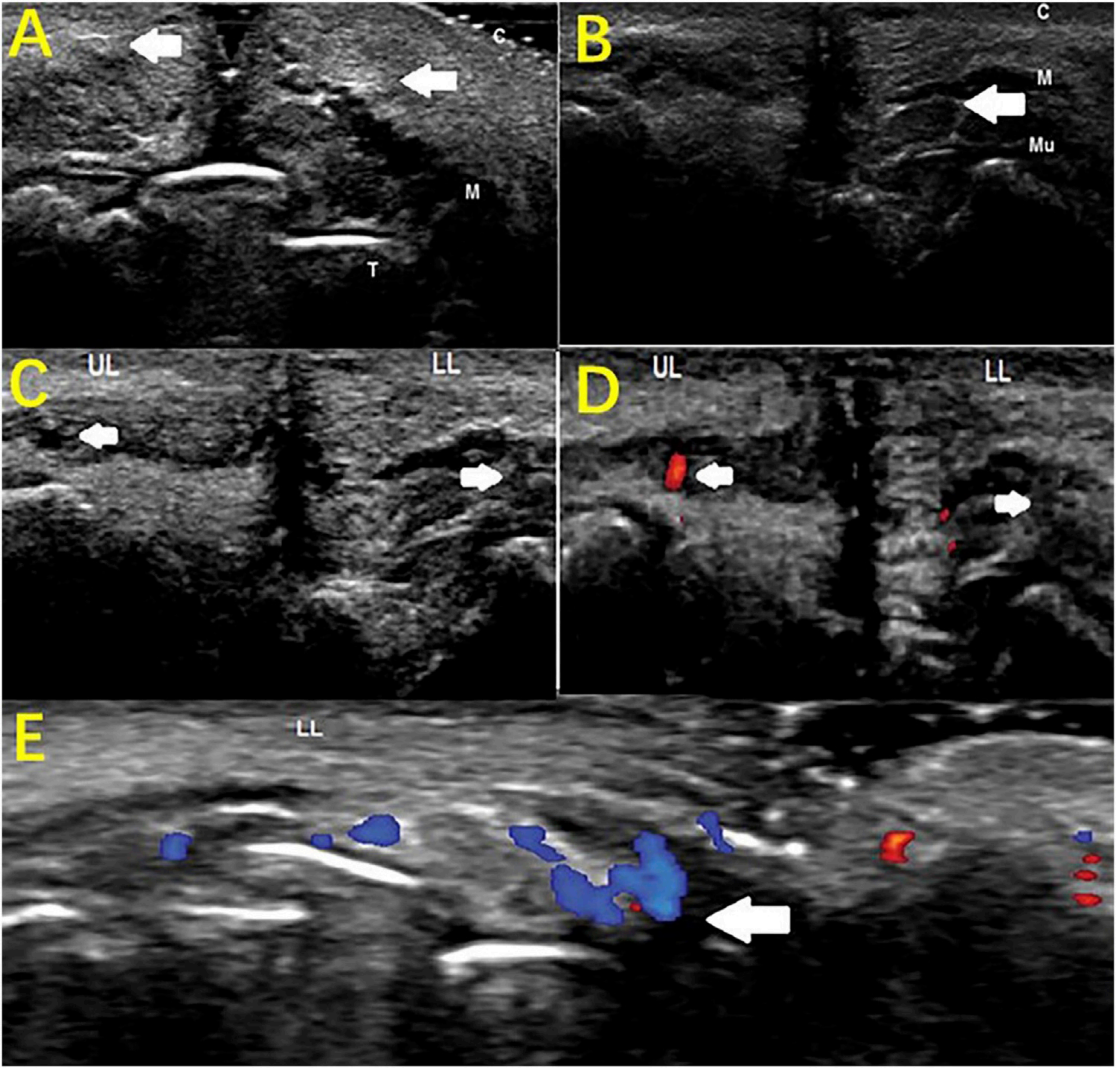
Monitoring complications of esthetic treatments by HFUS imaging. Sagittal plane **(A)** Hyperechoic lips (arrows); **(B)** Hypoechogenic area (filler deposit, arrow) surrounded by a hyperechoic region (T-teeth, M-orbicularis muscle, C-cutaneous tissue, Mu-mucosa). Sagittal plane **(C)** B-mode sonogram: The superior labial artery (left arrow), invisible inferior artery (right arrow); **(D)** Color Doppler sonogram: visible blood flow of the superior labial artery (left arrow), invisible blood flow of the inferior labial artery (right arrow); (LL: lower lip; UL: upper lip). Transverse plane **(E)** Color Doppler sonogram: collateral circulation of the left inferior labial artery (arrow).

**FIGURE 8:**
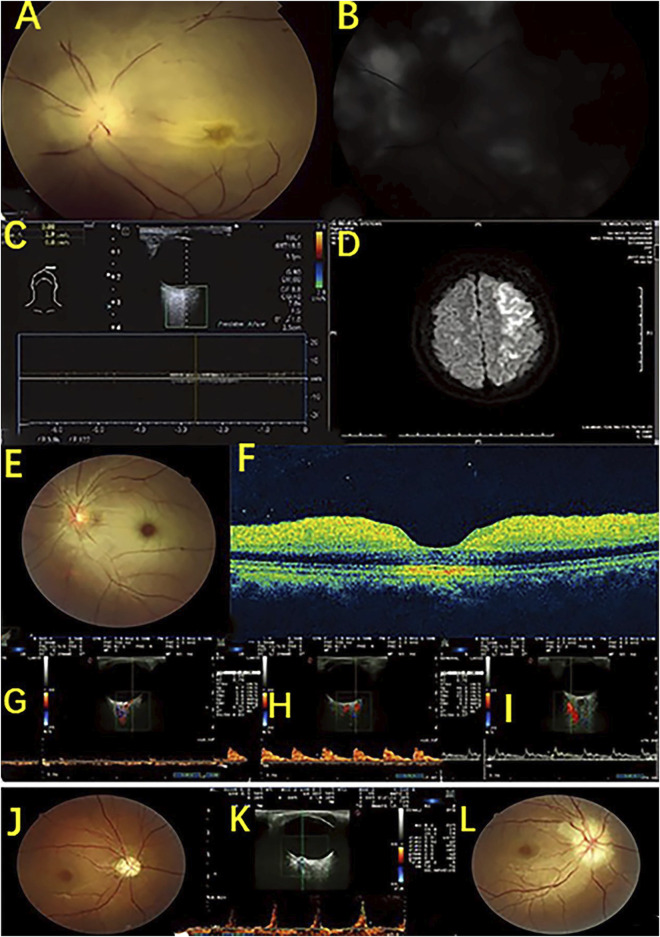
HFUS detection of retroocular artery occlusions resulting from cosmetic facial filler injections. Case 1. An ophthalmic artery occlusion caused by a cosmetic autologous fat injection. **(A)** Fundus photograph displaying diffuse retina edema and segmented retinal arteries; **(B)** Fundus fluorescein angiography showing severe damage to choroidal and retinal filling; **(C)** CDFI displaying no retrobulbar blood flow signal; **(D)** Diffusion weighted image showing a large area of acute infarction in the left posterior temporal lobe and occipital lobe. Case 2. A case of central retinal artery occlusion caused by a cosmetic autologous fat injection. **(E)** Fundus photograph image showing retinal whitening with a cherry red spot. **(F)** Optical coherence tomography image indicating inner retinal edema. **(G–I)** CDFI shows no retrobulbar blood flow signal in the central retinal artery, a slight reduction in the posterior ciliary arteries, and a normal blood flow signal in the ophthalmic artery. Case 3. A case of anterior ischemic optic neuropathy after a hyaluronic acid injection. A fundus showing optic disc edema at baseline **(J)** and pale optic disc at follow-up **(L)**; **(K)** CDFI showing a decreased end diastolic velocity and an increased resistance index and pulsatility index of the central retinal artery and a high peak velocity of arterial blood.

HFUS can also be used to monitor for complications from beauty treatments. Mlosek et al. (2019) reported a case of lip asymmetry, showing “lumps” in the lips as well as sensory and motor impairments in a 43-year-old woman 8 months after lip augmentation. HFUS found enhanced soft tissue echoes in the lips, deep filler injections, and positive signs, such as closed arteries under the lips, suggesting that HFUS can help diagnose complications of the lips (Figure 7). Doppler blood flow imaging is a logical addition to HFUS. Huang et al. (2019) used color doppler blood flow imaging (CDFI) based on HFUS to assess retrobulbar blood flow in 10 patients with closed eye arteries caused by the injection of facial fillers and explored the correlation between the retrobulbar ocular blood flow parameters and the clinical performance. The blood flow parameters differed in different parts and to varying degrees in the closed ball of the eye artery (Figure 8). When the patients had consciousness disturbances, fluorescent imaging or cerebrovascular angiography examinations were unavailable. Bedside CDFI based on HFUS was an available and practicable technique in this condition to detect the position of and extent of eye arterial closure.

### Limitations and Contraindications

Skin ultrasonic diagnostic equipment requires high frequency and may not accurately display the fine structure of skin when the ultrasound is below 20 MHz. Moreover, a probe of 50 MHz or above is needed to observe the epidermis more carefully. Skin ultrasound also has high technical requirements for the operators. For the skin ultrasound examination, a sufficient coupling should be ensured to obtain a satisfactory image quality. For open, infectious or fluid-contaminated lesions, the couplers cannot be used directly. The probe must be protected during the examination to prevent contamination of the probe. Despite these reports, there are generally no obvious contraindications for the use of HFUS imaging, and the clinical application value is still great. There is potential and practical value to using advanced emerging HFUS imaging as a scientific tool for cosmetic medicine imaging technology (Vergilio et al., 2021).

## Conclusion

HFUS is noninvasive, radiation-free, simple, and economical and is more commonly applied in the evaluation of skin diseases, such as scars, PWS, acne, and skin aging. Skin beauty treatment and efficacy evaluation play a very important auxiliary role, but the clinical application of HFUS in medical cosmetology is still at the initial stage. The main difficulty lies in thin-layered skin and the need for a longer learning curve by the skin HFUS operator. High levels of technical and professional knowledge by the operator are required. With growing attention given to skin-related HFUS imaging, an in-depth cooperation between dermatologists and ultrasound professionals based on the understanding of cosmetic pathology will greatly promote the application of HFUS in medical cosmetology, which can extend far beyond basic screening, and this will bring about convenient clinical recognition and promising diagnostic outcomes.
